# Development of a prediction model for neonatal hypoglycemia risk factors: a retrospective study

**DOI:** 10.3389/fendo.2023.1199628

**Published:** 2023-07-17

**Authors:** Tian Wu, Yi-Yan Huang, Wei Song, Sharon R. Redding, Wei-Peng Huang, Yan-Qiong Ouyang

**Affiliations:** ^1^ School of Nursing, Wuhan University, Wuhan, Hubei, China; ^2^ Department of Obstetrics, Wuhan Central Hospital, Tongji Medical College, Huazhong University of Science and Technology, Wuhan, Hubei, China; ^3^ Department of Nursing, Sir Run Run Shaw Hospital, Zhejiang University School of Medicine, Hangzhou, China; ^4^ Global Health of Project HOPE, Washington, MD, United States; ^5^ Department of Critical Care Medicine, Zhongnan Hospital of Wuhan University, Wuhan, Hubei, China; ^6^ Department of Critical Care Medicine, Sir Run Run Shaw Hospital, Zhejiang University School of Medicine, Hangzhou, China

**Keywords:** neonatal hypoglycemia, nomogram, predictive model, retrospective study, risk factors

## Abstract

**Background:**

It’s challenging for healthcare workers to detect neonatal hypoglycemia due to its rapid progression and lack of aura symptoms. This may lead to brain function impairment for the newborn, placing a significant care burden on the family and creating an economic burden for society. Tools for early diagnosis of neonatal hypoglycemia are lacking. This study aimed to identify newborns at high risk of developing neonatal hypoglycemia early by developing a risk prediction model.

**Methods:**

Using a retrospective design, pairs (470) of women and their newborns in a tertiary hospital from December 2021 to September 2022 were included in this study. Socio-demographic data and clinical data of mothers and newborns were collected. Univariate and multivariate logistic regression were used to screen optimized factors. A neonatal hypoglycemia risk nomogram was constructed using R software, and the calibration curve and receiver operator characteristic curve (ROC) was utilized to evaluate model performance.

**Results:**

Factors integrated into the prediction risk nomogram were maternal age (odds ratio [OR] =1.10, 95% CI: 1.04, 1.17), fasting period (OR=1.07, 95% CI: 1.03, 1.12), ritodrine use (OR=2.00, 95% CI: 1.05, 3.88), gestational diabetes mellitus (OR=2.13, 95% CI: 1.30, 3.50), gestational week (OR=0.80, 95% CI: 0.66, 0.96), fetal distress (OR=1.76, 95% CI: 1.11, 2.79) and neonatal body mass index (OR=1.50, 95% CI: 1.24, 1.84). The area under the curve (AUC) was 0.79 (95% confidence interval [CI]: 0.75, 0.82), specificity was 0.82, and sensitivity was 0.62.

**Conclusion:**

The prediction model of this study demonstrated good predictive performance. The development of the model identifies advancing maternal age, an extended fasting period before delivery, ritodrine use, gestational diabetes mellitus diagnosis, fetal distress diagnosis and an increase in neonatal body mass index increase the probability of developing neonatal hypoglycemia, while an extended gestational week reduces the probability of developing neonatal hypoglycemia.

## 1 Introduction

The fetus mainly obtains glucose from the mother and stabilizes its plasma glucose level. However, some newborns may develop hypoglycemia as they transit from intrauterine to extrauterine life. Neonatal hypoglycemia (NH) is a common neonatal metabolic disorder related to alterations in maternal and fetal metabolism, as well as disruptions in insulin production and function (1). The main clinical manifestations of NH are irritability, shortness of breath, decreased muscular tone, feeding difficulties, hypothermia, convulsion, or lethargy. Sometimes, neonatal hypoglycemia can be asymptomatic or accompanied by nonspecific manifestations (2). The incidence of NH is 10-20% (3–5), while in high-risk newborns, it can be as high as 50% (6). The incidence is still rising along with the increase of high-risk factors (7).

Due to the lack of specificity, diagnosis and intervention are difficult for healthcare providers, which may lead to newborns underwent continuous hypoglycemia, even irreversible brain injury and neurological dysfunction. In a follow-up study, 36% of newborns with NH had neurosensory impairment, including neurodevelopmental delay, visual impairment and behavioral problems (2, 8, 9). The separation of neonates with transitional hypoglycemia from their mothers to Neonatal Intensive Care Unit results in a great burden on both the family and society with family distress and increased medical costs (10, 11). Therefore, it is important to prevent its occurrence, minimize the risk factors and improve prenatal measures.

Previous studies have focused on the risk factors, management and prognosis of NH, the majority of which were focused on secondary prevention (12–15). An early identification tool to identify NH high-risk groups is lacking (6). This study aimed to develop a concise risk prediction model for early identification of NH by health professionals and to provide targeted management strategies to prevent NH.

## 2 Method

### 2.1 Study design and setting

Using a retrospective design, mothers and their newborns in a tertiary-level hospital from December 2021 to September 2022 in Wuhan, China were included in this study. Women were admitted to the hospital prior to labor or at the time of labor onset. Mothers and infants were discharged three to five days after delivery unless there were birth complications or other illnesses. Socio-demographic data and clinical data of mothers and newborns were recorded by the hospital information system (HIS) from admission to discharge. The study was reviewed and approved by the Ethics Committee of Wuhan University Medical Department, and the approval number is WHU-LFMD-IRB2023028.

### 2.2 Sample

To conduct multiple regression analyses, the sample size should be at least 15 times greater than the number of variables included (16). After conducting a thorough literature review and obtaining consensus among the researchers, a total of 26 variables were selected, encompassing socio-demographic data and clinical data (including previous medical history and complications) of mothers and newborns (**Table 1**) (17–21). Taking into account a potential attrition rate of 10%, a minimum of 429 pairs of mothers and newborns were required for the study.

**Table 1:** Socio-demographic characteristics of groups (N=470).

Characteristics	N (%)			
	Neonatal hypoglycemia (n=176)	Euglycemia (n=294)	Total (n=470)	*P* value
MATERNAL				
Maternal age (years)	31.00 (29.00 - 34.00)	30.00 (28.00 - 32.00)	30.00 (28.00-33.00)	<0.001
Employment				0.029
Unemployed	93 (52.8)	180 (61.2)	273 (58.1)	
Office clerk	49 (27.8)	80 (27.2)	129 (27.4)	
Private enterprise	19 (10.8)	12 (4.1)	31 (6.6)	
Technical staff	15 (8.5)	22 (7.5)	37 (7.9)	
Educational level				0.961
Junior high school	17 (9.7)	26 (8.8)	43 (9.1)	
Senior high school	17 (9.7)	29 (9.9)	46 (9.8)	
College degree	140 (79.5)	239 (81.3)	379 (80.6)	
Master’s degree	2 (1.1)	0 (0)	2 (0.4)	
MBMI (kg/m^2^)	27.55 ± 4.51	27.11 ± 4.39	27.35 ± 4.48	0.251
Delivery mode				<0.001
Vaginal	14 (8.0)	92 (31.3)	106 (22.6)	
Caesarean	162 (92.0)	202 (68.7)	364 (77.4)	
Gravidity	2.00 (1.00-3.00)	1.50 (1.00-2.00)	2.00 (1.00-3.00)	0.030
Parity	1.00 (1.00-2.00)	1.00 (1.00-2.00)	1.00 (1.00-2.00)	0.638
Fasting period (hours)	12.47 (10.61-14.99)	4.37 (0.00-13.89)	11.33 (0.00-14.33)	<0.001
Ritodrine use	34 (19.3)	28 (9.5)	62 (13.1)	0.002
Obesity	16 (9.1)	21 (7.1)	37 (7.9)	0.448
GDM	66 (37.5)	62 (21.2)	128 (27.2)	<0.001
Gestational week (weeks)	39.00 (38.00-39.00)	39.00 (39.00-40.00)	39.00 (38.00-40.00)	<0.001
Hypertension	10 (5.7)	28 (9.5)	38 (8.1)	0.139
Hepatitis B	7 (4.0)	13 (4.4)	20 (4.3)	0.817
Hypoproteinemia	16 (9.1)	17 (5.8)	33 (7.0)	0.174
Hyperlipidemia	35 (19.9)	40 (13.6)	75 (16.0)	0.072
PROM	7 (4.0)	33 (11.2)	40 (8.5)	0.009
Hypothyroidism	45 (25.6)	61 (20.7)	106 (22.6)	0.226
NEONATAL				
Gender				0.904
Female	92 (52.3)	152 (51.7)	244 (51.9)	
Male	84 (47.7)	142 (48.3)	226 (48.1)	
Fetal distress	73 (41.5)	86 (29.3)	159 (33.8)	0.007
Low birth weight	13 (7.4)	5 (1.7)	18 (3.8)	0.002
Macrosomia	12 (6.8)	10 (3.4)	22 (4.7)	0.090
Small for gestational age	4 (2.3)	16 (5.4)	20 (4.3)	0.099
Large for gestational age	49 (27.8)	39 (13.3)	88 (18.7)	<0.001
NBMI (kg/m^2^)	14.00 (13.28-14.80)	13.44 (12.62-14.27)	13.76 (12.79-14.42)	<0.001
Apgar	10.00 (10.00-10.00)	10.00 (10.00-10.00)	10.00 (0.00-10.00)	0.435

GDM, gestational diabetes mellitus; MBMI, maternal body mass index; NBMI, neonatal body mass index; PROM, premature rupture of membranes.

Small for gestational age, birthweight <10th percentile.

Large for gestational age, birthweight >90th percentile.

Inclusion criteria were newborns: (1) meet the diagnostic criteria for NH; (2) without malformations and developmental abnormalities. Exclusion criteria were: newborns or mothers with incomplete data.

Currently, the definition of NH remains controversial. The American Academy of Pediatrics recommended that measured by a laboratory enzymatic method, any newborn with blood glucose less than 40 mg/dL (2.22 mmol/L) within four hours of birth should be considered as having NH and necessitate medical intervention (22, 23). In this study, blood glucose was measured by a heel-stick on the newborn within 30 minutes of birth using a glucose oxidase electrode type A blood glucometer manufactured by Bayer.

### 2.3 Data collection

Data were collected from the HIS, including socio-demographic data and clinical data of mothers and newborns (see **Table 1**). As shown in **Table 1**, maternal body mass index (MBMI) was measured at admission, neonatal body mass index (NBMI) and the Apgar score were assessed at birth. The gravidity collected included this current pregnancy, while the parity did not. The fasting period was calculated from the start of fasting till the delivery of fetus. Based on the American Academy of Pediatrics (AAP) recommendations for interacting with neonates at risk of hypoglycemia (22), the following categories fall under this risk: low birth weight (LBW), which is defined as a birth weight equal to or less than 2500 grams, macrosomia that is defined as a birth weight equal to or greater than 4000 grams, small for gestational age (SGA) which is defined as a birth weight less than or equal to the 10th percentile, and finally large for gestational age (LGA) which is defined as a birth weight greater or equal to the 90th percentile.

A standardized data checklist was developed before data collection by the research team. Investigators reviewed the delivery record in the ward, and then filled the maternal and neonatal data in the checklist. Two investigators gathered the data independently and crosschecked each other’s work. Any disagreement about the data was resolved by checking the records with a third researcher.

### 2.4 Data analysis

Continuous variables were expressed by mean ± standard deviation (M ± SD) or median with interquartile range, and the discontinuous variables data were expressed as absolute numbers with percentages. For maternal and neonatal data, the Kolmogorov-Smirnov test was used to evaluate data normality.

Data analysis was conducted using SPSS (version 26.0) and R software (version 3.6.0). The process was divided into three stages: factors screening, model development and model evaluation. Using “glmnet” R packages, logistic regression analysis was used to identify risk factors from the 26 factors. Only variables with a P-value less than 0.05 in the univariate analysis were included in the multivariate regression analysis, and only variables with a P-value less than 0.05 in the multivariate analysis were selected for model development. Variables screened were entered into the model using “rms” R packages, allowing the nomogram to emerge. Using “rms” and “pROC” R packages, the calibration curve and the receiver operator characteristic curve (ROC) were depicted to evaluate the consistency and discrimination of the model.

## 3 Results

### 3.1 Socio-demographic characteristics

**Figure 1:**
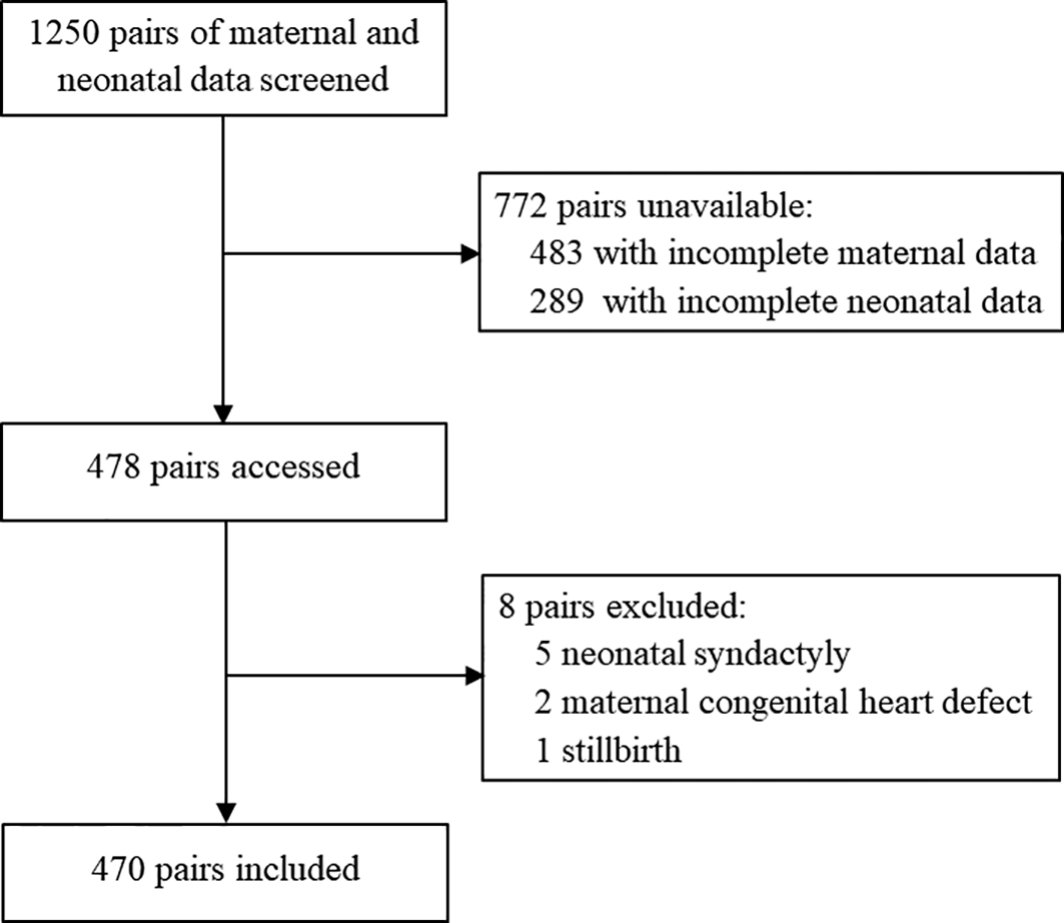
Flow chart of participant inclusion.

The participants’ inclusion process is shown in **Figure 1**. Of 1,250 mothers and newborns identified in HIS, 478 pairs were screened. Eight pairs were excluded due to congenital heart disease (two mothers), syndactyly (five infants) and stillbirth (one infant). A total of 470 pairs were eligible for participation and 176 (37.4%) infants had experienced NH.

Socio-demographic characteristics of participants are shown in **Table 1**. The maternal age was [30.00 (28.00-33.00)] years, and 55 participants (11.7%) were elderly parturient women. Forty-seven percent (221) were pregnant for the first time. Most of the infants (364, 77.4%) were delivered by cesarean birth, and about half of them (244, 51.9%) were female. The gestational week was [39.00 (38.00-40.00)] weeks.

### 3.2 Screening for predictive factors

**Figure 2:**
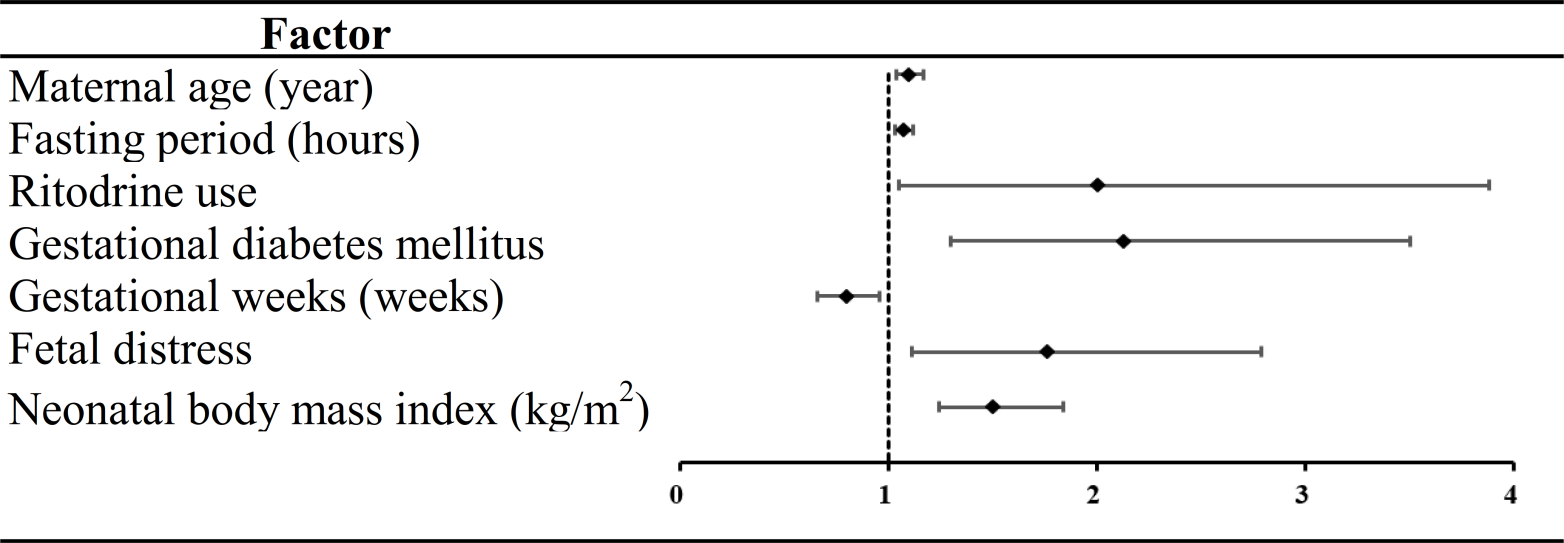
Forest plot of neonatal hypoglycemia.

Thirteen variables were found to be statistically significant using univariate analysis (*P <*0.05) (**Table 2**). Seven of the 13 factors showed statistically significant differences employed by multivariate analysis. Of these predictors, maternal age (odds ratio [OR] =1.10, 95% CI: 1.04, 1.17), fasting period (OR=1.07, 95% CI: 1.03, 1.12), ritodrine use (OR=2.00, 95% CI: 1.05, 3.88), gestational diabetes mellitus (GDM) (OR=2.13, 95% CI: 1.30, 3.50), fetal distress (OR=1.76, 95% CI: 1.11, 2.79) and NBMI (OR=1.50, 95% CI: 1.24, 1.84) were risk factors for NH. Gestational week (GW) (OR=0.80, 95% CI: 0.66, 0.96) was a protective factor for NH (**Figure 2**).

**Table 2:** Univariate and multivariate analysis of the influencing factors for neonatal hypoglycemia.

Variables	Univariate analysis		Multivariate analysis		
	OR (95% CI)	P	β	OR (95% CI)	P
Intercept			-2.35	0.09 (0.00, 177.19)	0.541
Maternal age	1.12 (1.06, 1.17)	<0.001	0.10	1.10 (1.04, 1.17)	0.002
Employment	1.22 (0.99, 1.50)	0.049	0.13	1.14 (0.90, 1.45)	0.256
Delivery mode	5.27 (2.98, 9.97)	<0.001	0.51	1.66 (0.76, 3.76)	0.205
Gravity	1.23 (1.03, 1.47)	0.025	0.01	1.01 (0.81, 1.26)	0.937
Fasting period	1.10 (1.07, 1.13)	<0.001	0.07	1.07 (1.03, 1.12)	<0.001
Ritodrine use	2.28 (1.33, 3.93)	0.003	0.70	2.00 (1.05, 3.88)	0.037
Gestational diabetes mellitus	2.25 (1.48, 3.40)	<0.001	0.77	2.13 (1.30, 3.50)	0.003
Gestational week	0.72 (0.61, 0.83)	<0.001	-0.23	0.80 (0.66, 0.96)	0.018
Premature rupture of membranes	0.33 (0.13, 0.72)	0.009	-0.97	0.37 (0.13, 1.02)	0.053
Fetal distress	1.71 (1.16, 2.54)	0.007	0.56	1.76 (1.11, 2.79)	0.016
Neonatal body mass index	1.54 (1.33, 1.80)	<0.001	0.41	1.50 (1.24, 1.84)	<0.001
Low birth weight	4.61 (1.71, 14.58)	0.004	1.16	3.19 (0.98, 11.72)	0.061
Large for gestational age	2.52 (1.58, 4.06)	<0.001	0.16	1.18 (0.64, 2.15)	0.598

CI, confidence interval.

β is the regression coefficient.

### 3.3 Development of prediction model

**Figure 3:**
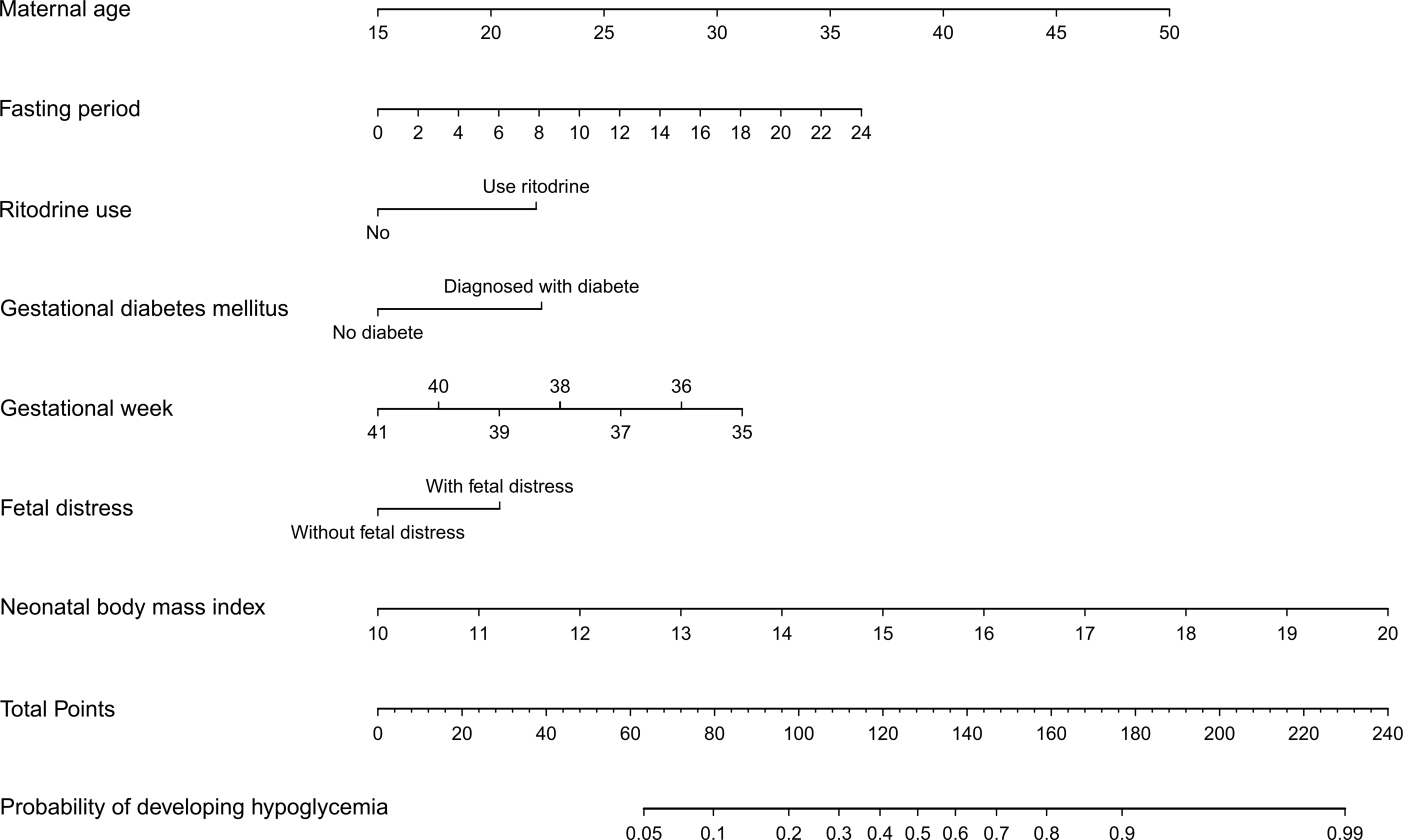
Prediction nomogram for neonatal hypoglycemia.

A risk nomogram was developed using the model that included all seven predictors, and it is a concise tool to predict the probability of developing NH. As shown in **Figure 3**, the nomogram comprises three parts, including the predictive factors, points and predicted probabilities. The top line is the point reference line for each factor, and the penultimate line is the total points of all the factors. To calculate the risk of developing NH, a vertical line is drawn for each predictor, with the vertical line corresponds to the points from the top line. The relevant scores are then summed to determine the total points (the penultimate line), which corresponded to the probability of experiencing NH (bottom line).

**Figure 4:**
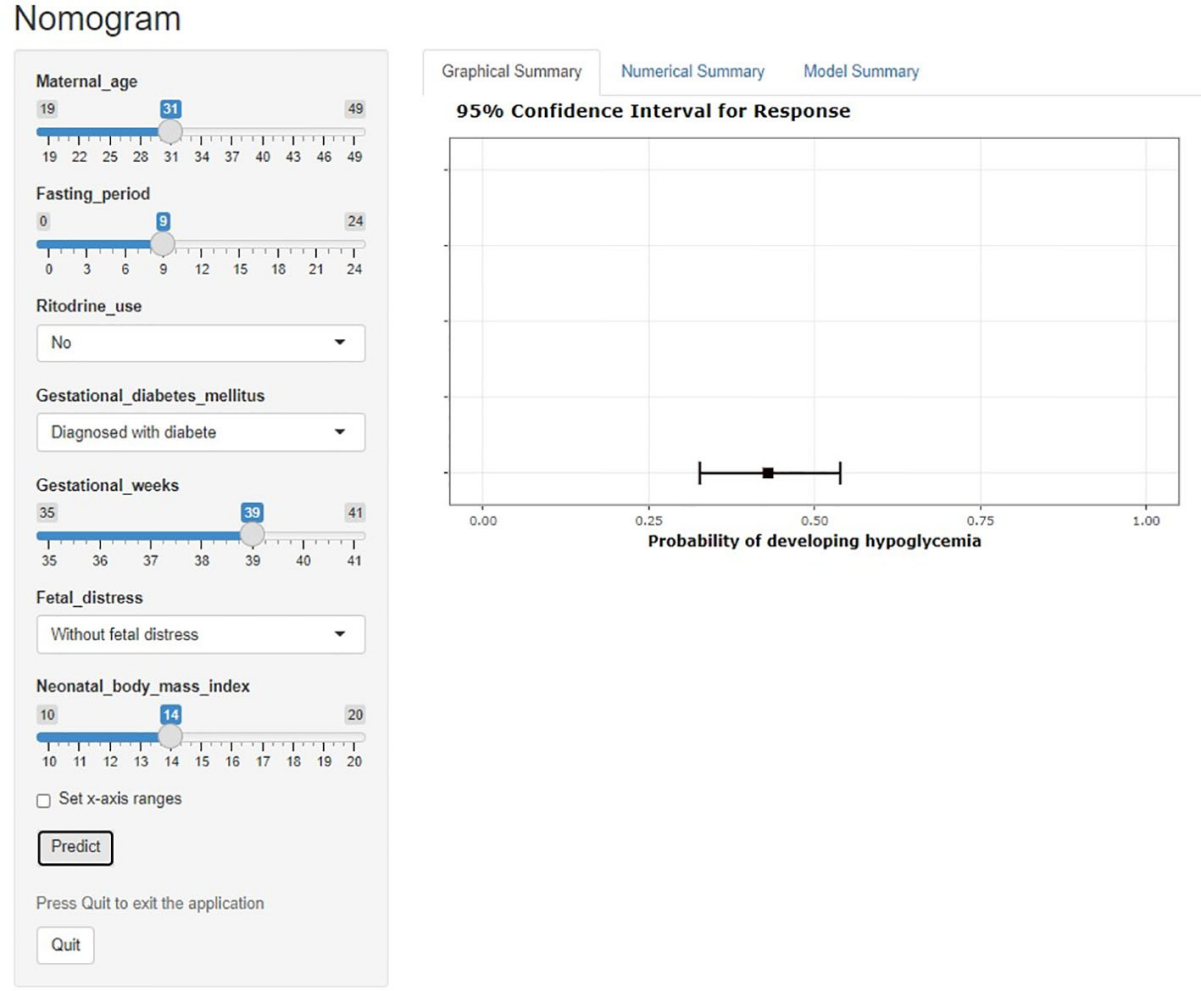
Dynamic nomogram for neonatal hypoglycemia.

A dynamic nomogram is intuitive and straightforward, requiring no manual computation. To obtain a predictive probability, healthcare providers need to input variables into the programmer, set x-axis ranges and click predict (**Figure 4**).

### 3.4 Evaluation of the prediction model

**Figure 5:**
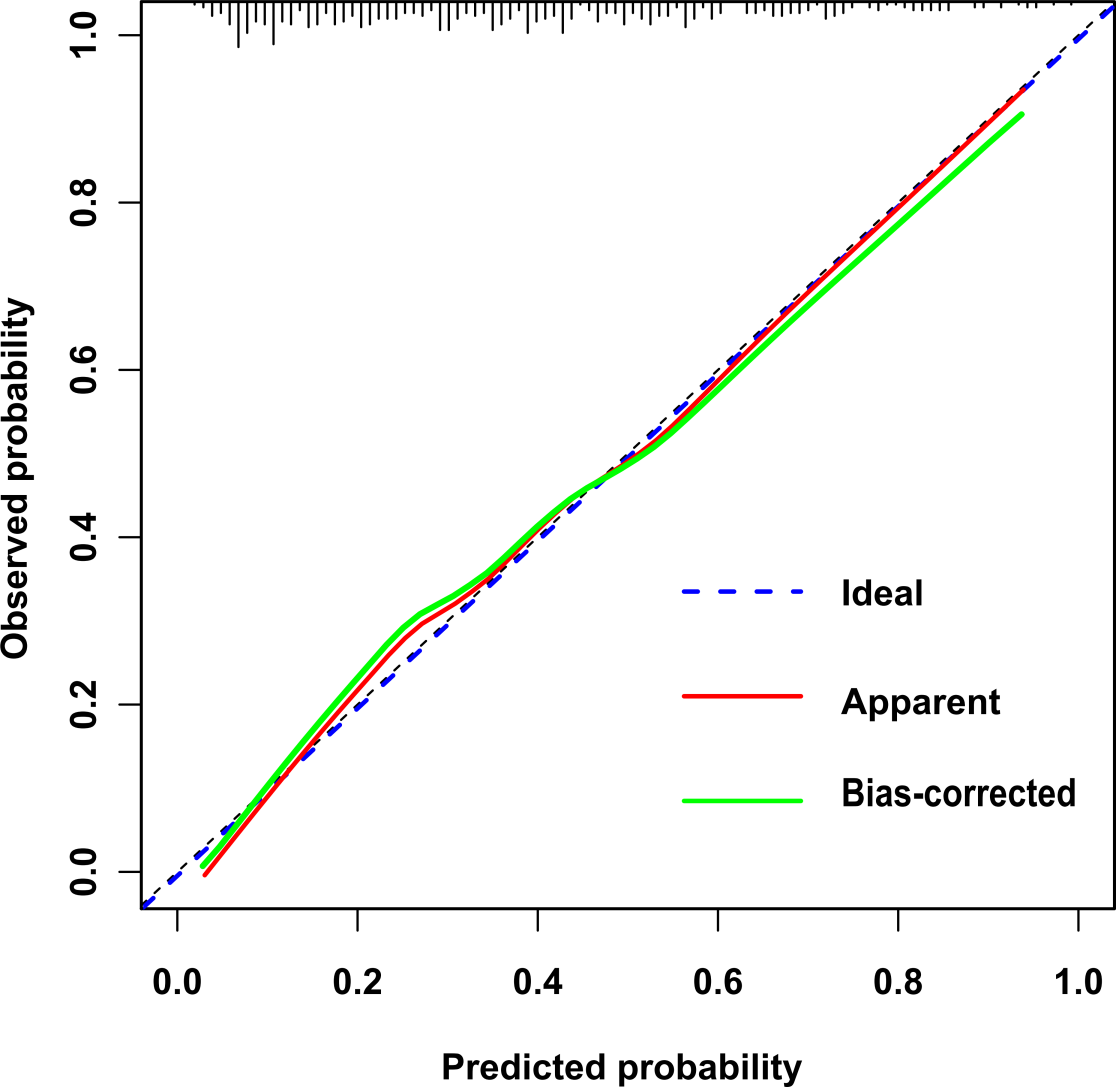
Calibration curve of nomogram.

**Figure 6:**
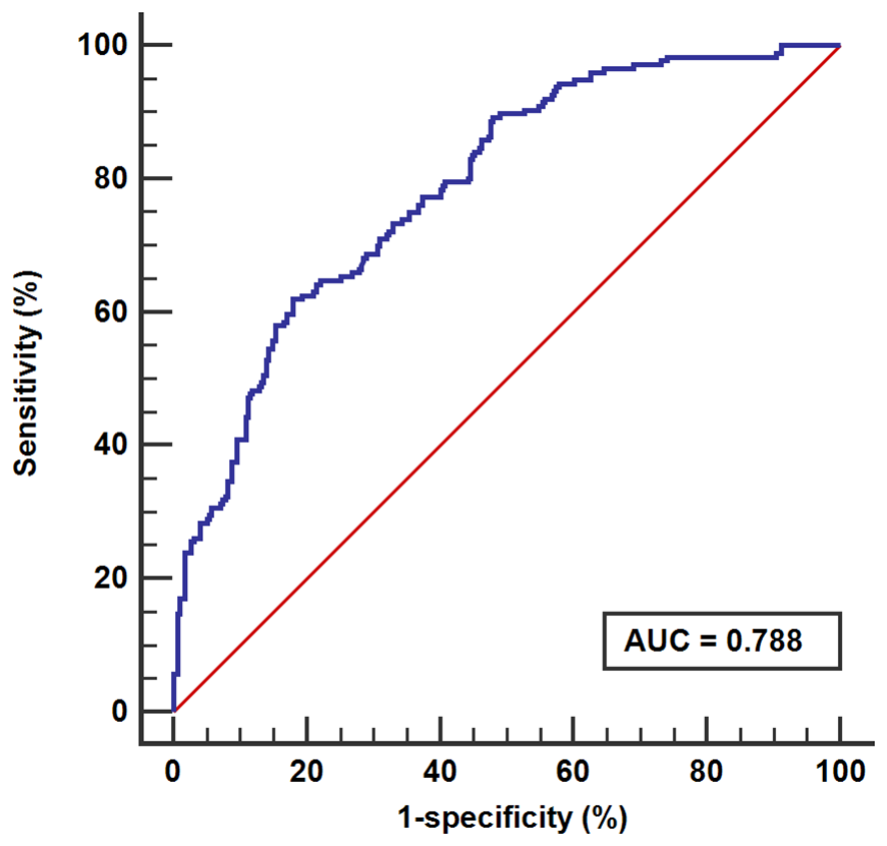
Receiver operating characteristic.

In this study, the calibration curve of the risk nomogram regarding prediction of NH showed good consistency (**Figure 5**). The uncorrected reference curve of the model is highly consistent and closely approximates the actual situation. The ROC curve was used to assess model differentiation and determine sensitivity and specificity, where the area under the ROC curve was 0.79 (95% CI: 0.75, 0.82). The specificity was 0.82, and the sensitivity was 0.62 (**Figure 6**).

## 4 Discussion

### 4.1 Predictors from the neonatal hypoglycemia risk nomogram

In this study, univariate analysis revealed 13 significant predictors out of 26 variables, while only seven predictors ultimately qualified for inclusion in the prediction model using multivariate analysis. The findings of the model fit and performance evaluation are in line with what was anticipated and show good predictive performance. Previous studies investigated risk factors of NH, but most of them only focused on a few or a single factor. This study developed a novel nomogram of NH for healthcare providers’ use in primary prevention.

To evaluate the risk of maternal age accurately, it was defined as a continuous variable rather than a binary variable in this research. The results of this article show that maternal age is a risk factor for NH, there have no studies reported an association between maternal age and NH. However, a meta-analysis showed a strong positive relationship between maternal age and GDM risk (24), especially for women aged 35 years and older (25). GDM is an independent risk factor for NH (26–28). It happens as a result of GDM have been shown to increase insulin resistance and fetal hyperinsulinemia in reaction to the mother’s uterine hyperglycemia (29). Therefore, it is necessary to pay attention to advanced maternal age. Having regular prenatal check-ups can also help to detect abnormal conditions early for further intervention. Maintaining stable blood glucose and body mass index is vital for them.

This study demonstrates that the risk of NH increases as the maternal fasting period lengthens, which is consistent with a previous study (30). Li et al. found that the preoperative fasting of two hours for clear fluids and six to eight hours for solid food not only reduced the incidence of NH but also reduced the incidence of vomiting. When fasting, some metabolic pathways work together to maintain normal blood glucose concentrations, which are regulated by hormones and autonomic nerves. In pregnancy, hormone changes that induce abnormal glucose metabolism may lead to maternal hypoglycemia, resulting in NH. Fasting is required for women undergoing cesarean delivery to prevent gastric reflux and aspiration caused by anesthesia. As the Enhanced Recovery After Surgery Guidelines recommended, two-hour fasting for liquids and a small meal six hours prior to cesarean delivery is appropriate, but in clinical practice the period is eight hours (31). Hence, limiting the duration of fasting is a way to maintain high glucose, especially for some women with metabolic diseases.

Belonging to the class of drugs known as β2-adrenergic receptor agonists, ritodrine’s primary mechanism of action is to stimulate β2-receptors on smooth muscles in the uterus, leading to relaxation and reduced contractions. However, its use has been linked to an increased risk of neonatal hypoglycemia due to its ability to inhibit insulin secretion from pancreatic beta cells, which may result in decreased glucose uptake by tissues (32, 33). Therefore, when considering the use of ritodrine during labor and delivery, it is critical to weigh its potential risks against benefits and make informed clinical decisions.

As one of the predictors, women with GDM were prone to give birth to infants with hypoglycemia due to abnormal fetal glucose and insulin homeostasis. This is consistent with the finding of a study that the risk of developing hypoglycemia increased 1.32-fold (95% CI: 1.25, 1.38, *P*<0.001) per gestational week at GDM diagnosis (34). Women with GDM produce more insulin than healthy pregnant women, maternal hyperglycemia stimulates fetal pancreatic beta cells to produce more insulin, which can lead to increased fetal nutrient uptake and growth. However, after birth, these infants are no longer exposed to the high glucose levels from their mothers, but continue to produce high levels of insulin, which can result in hypoglycemia. At present, the prevalence of GDM continues to rise globally, close monitoring of blood glucose levels in both mothers with GDM and their infants is essential to prevent and manage NH (28, 35, 36).

This study showed that the gestational week is a protective factor. Gestational week is negatively correlated with the risk of developing NH. This is consistent with a study that showed that premature infants are at higher risk of hypoglycemia than full-term infants (37). This may be due to insufficient glycogen and fat storage and reduced glucose formation in preterm infants. Hence, premature infants should be a focus of NH prevention.

Premature infants are at a high risk of developing NH due to their immature glucose-regulating mechanisms and limited glycogen stores (38). Close monitoring of blood glucose levels can help prevent and manage NH in premature infants. Breast milk provides the optimal source of nutrition for infants, and initiating breastfeeding within 30 to 60 minutes of birth, as advised by the Academy of Breastfeeding Medicine (23), can help prevent NH. Additional strategies hypoglycemia includes administering intravenous glucose solutions to women before or during childbirth and providing oral glucose and electrolyte supplements. Moreover, reducing the fasting period for mothers with premature infants can aid in glucose storage and promote speedy recuperation after delivery.

As for neonatal factors, infants who experienced fetal distress were 1.76 times more likely to develop NH as healthy infants (95% CI: 1.11, 2.79, *P*=0.016). Fetal distress triggers the release of stress hormones such as cortisol, factors contributing to hypoglycemia in “stressed” or SGA infants may include inadequacy of caloric/energy intake relative to energy expenditure, depletion of hepatic glycogen stores, defective gluconeogenesis, increased sensitivity to insulin, or adrenocortical insufficiency. The pathogenesis of transient hyperinsulinemic hypoglycemia in SGA infants is poorly understood but may be related in part to suppression of fetal insulin secretion by premature induction of cortisol and/or catecholamines (39, 40).

Infants with low body mass index were 1.50 times more likely to develop NH as healthy infants (95% CI: 1.24, 1.84, *P*<0.001). As an indicator to evaluate body proportion and nutritional status of newborns, NBMI can be calculated by evaluating weight and length via ultrasound during pregnancy, and measured immediately after birth, providing a basis for NH risk assessment. NBMI does reflect nutrition status growth status of newborns to some extent although in SGA and LGA infants were not found statistically significant in this model.

In this study, SGA was found not to be associated with the development of NH and LGA was significant only in the univariate analysis. It was consistent with the results of a cohort study, which showed that the probability of hypoglycemia was similar or even lower in SGA and LGA infants, or absolute high or low-weight infants than in infants without these risk factors (18). On the contrary, another previous study indicated that SGA was associated with a higher possibility of developing hypoglycemia (41). However, LGA was also mentioned as a controversial risk factor for NH in a clinical report (22). Mainly because it is challenging to rule out maternal diabetes or hyperglycemia. NH most frequently occurs in infants with impaired glucogenesis or ketogenesis, which may occur with excessive insulin production, altered production of counterregulatory hormones, a lack of substrate supply, or a disorder of fatty acid oxidation. Plasma glucose homeostasis requires glucogenesis and ketogenesis to maintain normal rates of fuel use. One of the reasons for no association with gestational age may be that infants can absorb adequate nutrition from the diet of mothers.

In the logistic regression analysis, delivery mode, maternal obesity, and hepatitis B were not found to have a significant impact. This finding is consistent with previous studies (37). However, there is ongoing debate regarding the relationship between maternal obesity and NH, with some evidence suggesting a correlation in a multicenter study (42). This relationship may be influenced by the limited sample size, highlighting the need for future research involving a larger cohort to investigate this further.

This study has limitations. Feeding status and birth temperature may be influencing factors of NH. Newborns who experience delayed or inadequate breastfeeding after birth are at high risk of developing NH, as the infant’s glucose reserves deplete rapidly without timely replenishment. However, kangaroo-mother care is now widely practiced in the clinical settings, with infants being carried by their mothers with skin-to-skin contact for warmth, breastfeeding, safety, and affection. This is one reason why the study did not include feeding condition and birth temperature as variables (43). In this study, we used a blood glucose meter to measure blood glucose, which is convenient and economical. However, its accuracy at low blood glucose levels is controversial (44, 45). Additional multi-centric studies in diverse populations are needed.

### 4.2 Predictive ability of the neonatal hypoglycemia risk nomogram

Considering the elevated prevalence, heightened risk, and asymptomatic manifestation of NH (46), it is particularly important to establish clinical prediction models for both patients and healthcare providers. The potency of a prediction model hinges on its ability to precisely forecast the development of illnesses, identify high-risk cohorts with precision, and offer a benchmark for clinical intervention that helps mitigate the likelihood of disease onset.

The ROC curve was used to assess the sensitivity and specificity of the model, and the AUC evaluated its consistency. Discrimination is defined as low (AUC between 0.5~0.7), good (AUC>0.7) and excellent (AUC>0.9) (47). In this study, the AUC was 0.79 (specificity=0.82; sensitivity=0.62), suggesting that the model has good predictive ability.

Early identify high-risk newborns can help to prevent the incidence of NH and related complications (48). A nomogram is a statistical model that is built on several variables. To deliver a more personalized diagnosis or prognosis, nomograms rely on user-friendly digital interfaces, greater accuracy and more readily comprehensible prognoses. This nomogram is a tool for healthcare providers to identify early and intervene with infants at high risk of developing NH before delivery, to reduce the incidence of NH and its series of complications, therefore reducing the family care burden and socio-economic burden. The dynamic nomogram proposed in this study does not require manual calculation of the probability of the disease, only the input of specific variables and the calculation of the probability of the event. The development of the nomogram identifies predictors and provides new insights into the prediction and primary prevention of NH.

## 5 Conclusions

In conclusion, the advancing maternal age, an extended fasting period before delivery, the use of ritodrine, diagnosis of GDM, diagnosis of fetal distress, and an increase in NBMI increase the probability of developing NH. Conversely, an extended GW reduces the probability of developing NH.

## Data availability statement

The original contributions presented in the study are included in the article/supplementary material. Further inquiries can be directed to the corresponding authors.

## Ethics statement

The study was reviewed and approved by the Ethics Committee of Wuhan University Medical Department, and the approval number is WHU-LFMD-IRB2023028. Written informed consent was obtained from the participant's legal guardian/next of kin.

## Author contributions

Y-QO and W-PH is the corresponding author. TW and Y-YH contributed equally and share the first authorship. Y-QO and W-PH conceived the study. TW and Y-YH collected, analyzed the data and wrote the original draft of the manuscript. WS collected the data and provided support, and SR revised and edited the article. All authors contributed to the article and approved the submitted version.
